# 45, X/ 46, X, psu idic (Y) (q11.2) Mosaicism in a Primary Amenorrhea Girl with Swyer Syndrome

**DOI:** 10.1155/2023/9127512

**Published:** 2023-03-09

**Authors:** Yu Han, Jiebin Wu, Fangfang Tan, Jing Sha, Bei Zhang, Jingfang Zhai, Xuezhen Wang

**Affiliations:** ^1^Graduate School of Xuzhou Medical University, Huaihai West Road No. 84, Xuzhou, Jiangsu, China; ^2^Department of Prenatal Diagnosis Medical Center, Xuzhou Central Hospital, Xuzhou Clinical School of Xuzhou Medical University, Jiefang South Road No. 199, Xuzhou, Jiangsu, China; ^3^Department of Gynecology and Obstetrics, Pizhou People's Hospital, Nanjing Road No. 9, Pizhou, Jiangsu, China; ^4^Graduate School of Bengbu Medical College, Donghai Avenue No. 2600, Bengbu, Anhui, China

## Abstract

The female characters with a 46, XY karyotype, historically termed Swyer syndrome, are commonly divided into complete and partial gonadal dysgenesis. The former is completely made up of the 46, XY chromosome, while the latter results from 45, X/46, XY mosaicism. Both of them are sex chromosome disorders and are typically characterized by delayed puberty and primary amenorrhea due to disruption of the embryonic gonads into testes. In this report, we described a young female with mos 45, X [2]/46, X, psu idic (Y) (q11.2) [48] by karyotyping. Further copy number variation sequencing (CNV-seq) and fluorescent in situ hybridization (FISH) verified her chromosome alteration. The following gonadectomy and hormone replacement therapy were carried out, and the menstrual cycle recovered along with the development of bilateral breasts and uteruses. Herein, we aim to provide clinical management strategies for the patient with Swyer syndrome in clinical practice.

## 1. Introduction

Swyer syndrome, which was put forward by Swyer in 1959, presents 46, XY female sex reversal syndrome manifestations, such as primary amenorrhoea and breast dysplasias, while the external genitalia show normal female manifestations with a prevalence of approximately one per 80,000 births [1]. As is well known, sex-determining region Y (SRY) (OMIM 480000) is a critical gene encoding a testis-determining factor (TDF), which inhabits Yp11.32 and determines a young man's normal male appearance. Individuals with a 46, XY karyotype showing phenotypical female characters have been confirmed to be related to the absence of the SRY gene [2]. However, in our report, the presence of the SRY gene, confirmed by FISH, led us to a dilemma: why did she present as a phenotypical female? We have reviewed relevant reports from the literature and further clarified the associated questions with Swyer syndrome.

## 2. Case Presentation

A 16-year-old girl (social gender) was referred to the gynecology department of Xuzhou Central Hospital due to primary amenorrhea in December 2017. She was short in stature and healthy phenotypically, with a height of 147 cm (<third percentile) and a weight of 45 kg (BMI: 20.8 kg/m^2^). The physical examination showed bilateral flat breasts and normal external genitalia, while her perioral hair was slightly thick. Endocrine analysis showed elevated serum hormone levels: luteinizing hormone (LH) 36.0 mIU/ml (normal range [NR]: 2.12–10.89) and follicle-stimulating hormone (FSH) 58.83 mIU/ml (NR: 3.85–8.78), and reduced serum hormone levels: estradiol 4.0 pg/ml (NR: 1.5–195) and anti-Mullerian hormone (AMH) 0.01 ng/ml (NR: 0.96–13.34), and the serum cortisol result was normal. Pelvic ultrasonography revealed a primordial uterus (29 × 24 × 19 mm) and bilateral appendages that were undetected. The results of an X-ray absorptiometry scan showed low bone mineral density.

She underwent a gonadectomy in a tertiary maternity hospital in Shanghai in early 2018, and histopathological and immunohistochemical examinations confirmed bilateral ovarian gonadoblastoma and dysgerminoma without normal ovarian or testicular tissue, with bilateral sizes: 1.5 *∗* 0.8 *∗* 0.6 cm. After the surgery, she received persistent periodic hormone replacement therapy. Dynamic examination of blood hormone levels indicated that estradiol levels fluctuated within the normal range, while the levels of LH and FSH were slightly elevated. The last result of serum endocrine in May 2022 indicated LH 22.0 mIU/ml, FSH 58.83 mIU/ml, and estradiol 45.0 pg/ml. The development of bilateral breasts and the uterus has been improved, and the menstrual cycle has been recovered.

In February 2022, after the patient's informed consent form had been signed, a chromosome examination of peripheral blood lymphocytes was carried out for the girl. CNV-seq of peripheral blood lymphocytes was carried out according to the laboratory's standard operating procedure. DNA extracted from the samples was detected at the whole genome level using the NextSeq CN500 gene sequencer (Illumina, uniq reads ≥2.5 Mb). Chromosome Analysis Suite (ChAS) software 3.1 was used to analyse the data. The gene copy number variants detected by CNV-Seq were evaluated by referring to the Human Genome version 19 (hg19), the DECIPHER database, and the OMIM database. FISH on the metaphase chromosomes of cultured blood lymphocytes was combined with DAPI banding analysis, using centromeric probe DXZ1 (Xp11.1-q11.1) and an SRY-specific probe (SRY, Yp11.3), following the instructions (FISH probes from Abbott U.S.). The results of chromosome analysis were named according to the International System for Human Cytogenomic Nomenclature (2020) (ISCN 2020) [3].

**Figure 1:**
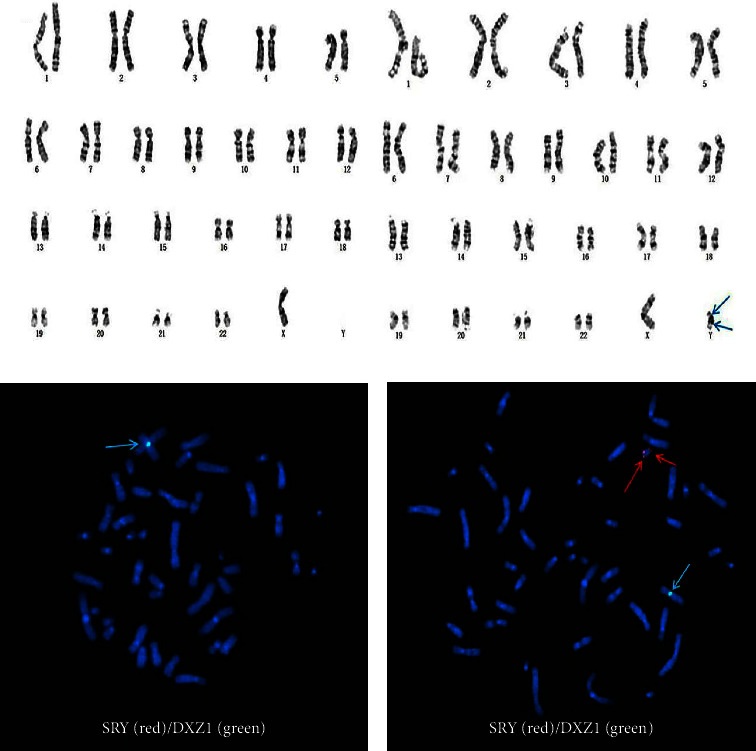
G-banding karyotype (a, b) combined with FISH (c, d) of the young girl's peripheral blood: mos 45, X [3]/46, X, ish psu idic (Y) (q11.2) (DXZ1+) (SRY++) [37]; (b) blue arrows indicated two centromere regions. (c, d): green arrows indicated X chromosome centromeric signals, and red arrows showed two SRY-specific signals on the two distal tips of the long arm of chromosome Y.

**Figure 2:**
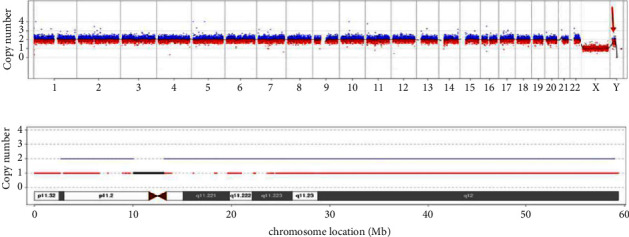
CNV-seq result: an approximately 59.37 Mb duplicated fragment in chrY: 1–59373566; in (b), the blue line represents the ratio curve and two copy numbers, corresponding to the left ordinate; the red line represents highly repetitive areas; the black line represents the region of the centromere. (a) Whole genome sequencing results. (b) chrY: 1–59373566.

G-banding karyotype analysis combined with metaphase FISH results: mos 45, X [3]/46, X, ish psu idic(Y) (q11.2) (DXZ1+) (SRY++) [37] (Figure 1) confirmed the presence of the SRY gene. The CNV-seq result illustrated an approximately 59.37 Mb duplicated fragment presented in chrY: 1–59373566 (Figure 2).

## 3. Discussion

**Figure 3:**
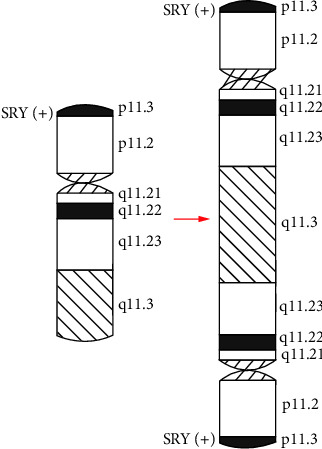
A diagram of the psu idic (Y).

Swyer syndrome is a kind of sex development disorder caused by a chromosomal abnormality. The individual in our report was currently diagnosed with Swyer syndrome based on primary amenorrhea, bilateral breast dysplasias, bilateral streak ovaries, changes in blood endocrine levels, and chromosomal results, which classified her as having as partial gonadal dysgenesis according to the karyotyping result. Like most patients with Swyer syndrome, this patient presented with primary amenorrhea and bilateral breast dysplasias because of blood hypoestrogenemia at the initial discovery of the disease [4]. However, the conditions of both estradiol, LH and FSH levels and breast development have improved after gonadectomy through postoperative hormone replacement therapy. Different from the traditional Swyer syndrome, her stature was short, which might be associated with 45, X/46, XY mosaicism [5] and low bone mass [6]. Gole et al. reported a female patient with mos 45, X/46, X, psu dic (Y) (q11.2) and whose stature was short too; however, her clitoris was enlarged, and one of her gonads was an ovotestis. The formation of an isodicentric Y chromosome might originate from a single break in one of the Y chromatids during the process of mitosis or meiosis, followed by a fusion of the broken ends of sister chromatids and frequently accompanied by the presence of 45, X, which might lead to Y chromosome fragment duplication [7] (Figure 3). In this report, G-banding and FISH confirmed the abnormal karyotype: mos 45, X [3]/46, X, ish psu idic (Y) (q11.2) (DXZ1+) (SRY ++) [37] (Figure 1), and CNV-seq also revealed an approximately 59.37 Mb fragment repeatedly presented in chrY: 1–59373566 (Figure 2). So far, we have no evidence to identify how the duplication of the fragment containing the SRY gene affects gene expression. As is known, the presence of male-to-female sex reversal is usually associated with the SRY gene, which encodes the testis-determining factor (TDF). However, in our case, G-banding karyotype combined with FISH result showed the mosaicism composed of 45, X and 46, X, psu idic (Y) (q11.2), where the lymphocytes of peripheral blood in our case showed 92.5% (37/40) of cells with the psu idic (Y) chromosome in which two SRY positive signals expressed, whereas the patient has been presenting a female appearance. To the best of our knowledge, this kind of karyotype has not been reported in Swyer syndrome, and the presence of the SRY gene is not a suitable indicator for the resulting phenotype in our case. Therefore, we shifted the focus to mosaicism: 45, X/46, X, psu idic (Y) (q11.2). And Gole et al. suggested that the Swyer phenotype in the presence of SRY may be due to skewed discrepancies in the percentages of the 45, X and the 46, X, der (Y) clones distributed between peripheral blood lymphocytes (PBL), the gonads, and other somatic tissues determining external female genitalia and other female characters [7]. Indeed, in their case, only 10% of the PBL were 45, X, while 90% displayed a 46, X, dic (Y) (q11.2) karyotype, whereas in the gonads the distribution was 45, X [69]/46, X, dic (Y) (q11.2) [31] (right gonad) and 45, X [100] (left gonad) [7]. For the moment, the 92.5% Y cells in the peripheral blood cannot also explain the female phenotype. Unfortunately, it is hard to gain tissue specimens from a prior gonadectomy in the original surgical hospital. Hence, we speculated on the possibility of a discrepant 45, X/46, X, der (Y) mosaicism status between the PBL, the gonads, and other somatic tissues in this female, requiring management by the full Turner syndrome protocols. In Turner syndrome, we should not only give hormone replacement therapy (including growth hormone and estrogen therapy) based on gonadal dysplasia and low bone mineral density but also pay attention to cardiovascular disease [8].

G-banding and FISH have revealed 45, X/46, X, idic (Y) mosaicism, while the histopathological analysis showed the presence of bilateral ovarian gonadoblastoma combined with dysgerminoma. It has been proven that 45, X/46, XY was obviously associated with a high risk of gonadoblastoma, and it is noted that female individuals with 45, X/46, XY mosaicism should be recommended to accept a gonadectomy [9]. In addition, postoperative hormone replacement therapy and clinical follow-up are also necessary. Bilateral gonadal dysgenesis or gonadoblastomas usually lead to a reduction of estrogen levels in this case and elevated gonadotropin in the feedback, which reminds clinicians to provide hormone replacement therapy for patients after the surgery. As for the young woman in this report, who accepted periodic hormone replacement treatment, the uterine development has been well improved. It is highly likely to have a child after receiving an egg donation.

## 4. Conclusion

In conclusion, genetic testing is an appropriate detection method for individuals with disorders of sex development and reproductive/pubertal differences. The diagnosis is readily established by genetic analysis combined with endocrine examination. Treatments through hormone replacement therapy or surgical therapy and frequent follow-up are essential for patients with Swyer. The mechanism of the relationship between psu idic (Y) (q11.2) and sex determination still has many possibilities to be further explored.

## Data Availability

The data of this study are available at the Department of Prenatal Diagnosis Medical Center, Xuzhou Central Hospital.
